# Impact of Short Sleep Duration on the Incidence of Obesity and Overweight among Children and Adolescents

**DOI:** 10.3390/medicina58081037

**Published:** 2022-08-02

**Authors:** Seung-Ho Han, Jae-Yong Yee, Jung-Soo Pyo

**Affiliations:** 1 Department of Physiology and Biophysics, Eulji University School of Medicine, Daejeon 34824, Korea; hans0424@eulji.ac.kr (S.-H.H.); jyee@eulji.ac.kr (J.-Y.Y.); 2 Department of Pathology, Uijeongbu Eulji Medical Center, Eulji University School of Medicine, Uijeongbu 11759, Korea

**Keywords:** sleep, obesity, overweight, body mass index, meta-analysis

## Abstract

*Background and Objectives*: The prevalence of obesity among children is increasing and is highlighting many problems. Lack of sleep is common among children and adolescents. Although several studies have investigated sleep duration and overweight and obesity from a sex perspective, data regarding age and sex effects remain limited and inconclusive. This study aimed to evaluate the risk(s) for overweight or obesity according to sleep duration among children and adolescents; to evaluate the effect of short sleep duration on the incidence of overweight/obesity among children and adolescents; and to evaluate sex differences in the risk of overweight or obesity with shorter sleep durations. *Materials and Methods*: The PubMed database was searched for relevant studies published up to June 30, 2021. Odds ratios for obesity/overweight were estimated for short compared with long sleep duration. Subgroup analysis based on sleep duration, sex, and study location was also performed. *Results*: The estimated odds ratio for combined obesity and overweight was 1.171 (95% confidence interval (CI) 1.092–1.256) according to short sleep duration. Obesity/overweight with short sleep duration was significantly prevalent in the <6 and 6–10 years’ subgroups (odds ratio 1.226 (95% CI 1.083–1.387) and 1.341 (95% CI 1.175–1.530), respectively). Among boys, short sleep duration was significantly correlated with a high occurrence of obesity/overweight (odds ratio 1.294 (95% CI 1.153–1.452)); no such correlation was found among girls. *Conclusions*: Short sleep duration may increase risk of obesity among children and adolescents, especially those <6 and 6–10 years of age. In the subgroup analysis, the incidence of obesity/overweight for short sleep time revealed significant results among Asians and boys.

## 1. Introduction

According to the World Health Organization, the global prevalence of obesity nearly tripled between 1975 and 2016. In 2016, 39% of adults ≥ 18 years of age were overweight, 13% were obese, and >340 million children and adolescents 5–19 years of age were overweight or obese. In 2020, 39 million children < 5 years of age were overweight or obese [1]. BMI is often used to measure the degree of obesity. BMI was calculated as weight in kilograms divided by height in meters squared. According to the World Health Organization (WHO) criteria, obesity is defined as a BMI ≥ 30 kg/m^2^ (normal weight < 25.0, overweight = 25.0–29.9, obesity ≥ 30 kg/m^2^).

The prevalence of obesity among children is increasing and, as a result, many problems are being highlighted. Overweight and obesity among children and adolescents contribute to the onset of chronic diseases, cognitive decline, and the onset of psychological disorders, such as mental stress and feelings of inferiority, which lowers self-esteem and leads to a passive attitude in interpersonal relationships [2,3]. This may adversely affect normal development into adulthood [3,4].

Humans devote a significant portion of the day to sleep. Sleep duration varies among individuals and with age. However, according to the American Academy of Sleep Medicine, for optimal health, children 3–5 years of age should sleep 10–13 h per day and those 6–12 years, 9–12 h and, for adolescents 13–18 years of age, 8–10 h is recommended [5]. Data from the United States demonstrated that the prevalence of short sleep duration among middle-school students was 57.8% and 72.7% among high school students [6]. In 2010, an epidemiological study involving primary and secondary students 9–18 years of age, from 30 provinces across China, reported that <39.09% of students reported having >8 h of sleep per night; the prevalence reached 93.64% of all students [7]. In modern society, lack of sleep is common among children and adolescents. Inadequate sleep among children and adolescents is associated with an increased risk of obesity, diabetes, injuries, poor mental health, attention and behavioral problems, and poor academic performance [8,9,10].

Men and women have different lifestyles, hormonal influences, and social concepts, suggesting the possibility of different gender-related effects on sleep patterns and obesity. Women may experience severe sleep problems as well as increases in obesity and waist circumference due to menopause, and there are objective sleep disturbances in elderly men and women [11,12].

Although several studies have investigated sleep duration and overweight and obesity from a sex perspective, data regarding age and sex effects remain limited and inconclusive [13,14,15,16]. Given this background, we aimed to account for adequate sleep duration among children and adolescents and to investigate whether short sleep duration is an independent risk factor for overweight and obesity and/or for sex- and age-specific effects. Despite its public health importance, there are few reports describing the association between overweight/obesity and short sleep duration across various age groups.

More specifically, the goals of the present study were to evaluate the risk(s) for overweight or obesity according to sleep duration among children and adolescents; to evaluate the effect of short sleep duration on the incidence of overweight/obesity among children and adolescents; and to evaluate sex differences in the risk of overweight or obesity with shorter sleep durations.

## 2. Materials and Methods

### 2.1. Literature Search and Study Selection Criteria

A literature search of the PubMed and MEDLINE databases for relevant studies published up to 30 June 2021 was performed. The database was searched using the following keywords: “Sleep duration”; “obesity or body weight or body mass index”; “children or adolescent”; and “prospective or cohort or observational”. The titles and abstracts of all retrieved articles were screened for eligibility. Review articles were also screened to identify additional potentially eligible studies. Studies involving children and/or adolescents and reporting information regarding obesity or overweight according to sleep duration were included. Case reports, nonoriginal articles, and those not published in English were excluded. This study was registered in RPOSPERO.

### 2.2. Data Extraction

Data were extracted from each eligible study by two researchers [15,16,17,18,19,20,21,22,23,24,25,26,27,28,29,30,31,32,33]. Extracted data included the following: first author’s name; year of publication; study location; number of children and adolescents analyzed; and the risk of obesity or overweight. To perform subgroup analysis, information regarding the risk of obesity and overweight according to sleep duration, sex, and study location was investigated.

### 2.3. Statistical Analyses

Meta-analysis was performed using the Comprehensive Meta-Analysis software package (Biostat, Englewood, NJ, USA). The odds ratios for obesity/overweight were estimated for short sleep duration compared with long sleep duration. Subgroup analysis according to sleep duration, sex, and study location was also performed. Heterogeneity among studies was assessed using the Q and I^2^ statistics and expressed as *p*-values. Additionally, sensitivity analysis was conducted to assess the heterogeneity of eligible studies and the impact of each study on the combined effect. In addition, to compare between boy and girl subgroups, the metaregression test was performed. Because eligible studies reported various sleep durations and differing populations, the application of the random-effect model, rather than the fixed-effect model, was more suitable. To assess publication bias, Begg’s funnel plot and Egger’s test were used. If significant publication bias was detected, the fail-safe N and trim-fill tests were additionally performed to confirm the degree of publication bias. The risk of bias was assessed through ROBINS-I tool. Differences with *p* < 0.05 were considered to be statistically significant.

## 3. Results

### 3.1. Selection and Characteristics of the Included Studies

**Figure 1:**
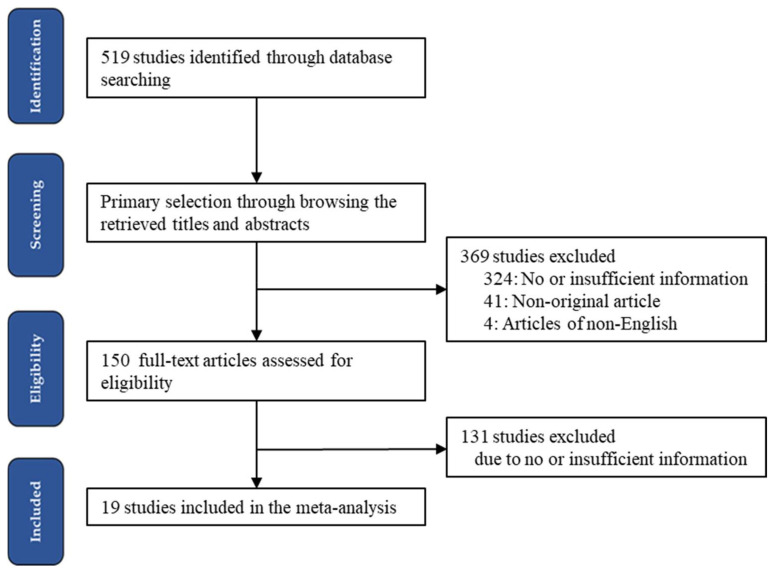
Flow diagram illustrating the study search and selection methods.

The initial literature search retrieved 519 articles from the database. On primary screening, 369 articles were excluded due to the absence of or insufficient information (*n* = 324), nonoriginal studies (*n* = 41), and non-English publications (*n* = 8). The full text of 150 articles was reviewed, with 131 excluded due to the absence of or insufficient information. Ultimately, therefore, 19 articles were included in the meta-analysis. Detailed information regarding the included and excluded studies is shown in Figure 1 and summarized in Table 1.

### 3.2. Estimated Risks for Obesity and Overweight According to Short Sleep Duration

The estimated odds ratio for combined obesity and overweight was 1.171 (95% confidence interval (CI) 1.092–1.256) according to short sleep duration in the overall cases (Table 2).

**Figure 2:**
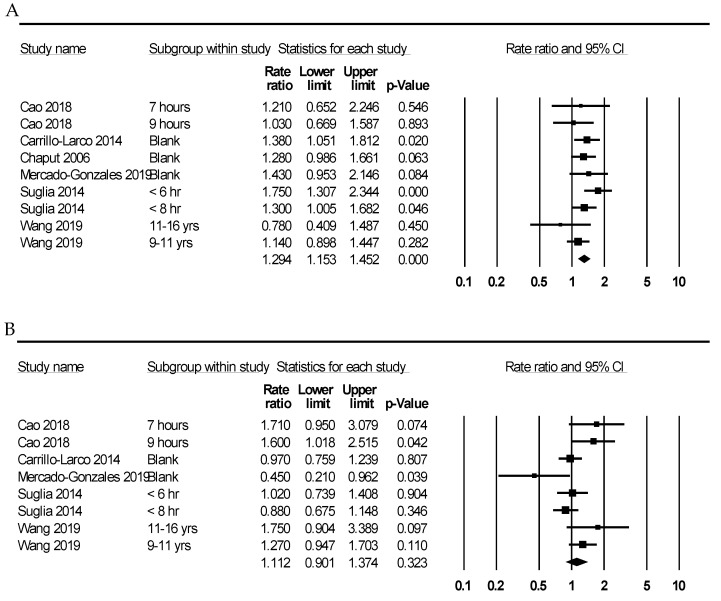
Forest plots of odds ratios for obesity/overweight according to short sleep duration in boys (**A**) and girls (**B**).

The odds ratios for obesity and overweight were 1.191 (95% CI 1.055–1.344) and 1.098 (95% CI 0.976–1.234), respectively. In the subgroup analysis based on age, obesity/overweight with short sleep duration was significantly prevalent in the <6 and 6–10 years’ subgroups (odds ratio 1.226 (95% CI 1.083–1.387) and 1.341 (95% CI 1.175–1.530), respectively). There was no significant difference between short and long sleep duration in the >10 years’ subgroup. A significant correlation between obesity/overweight and short sleep duration was found in the Asian but not in the North American subgroups (odds ratio 1.161 (95% CI 1.060–1.398) and 1.161 (95% CI 0.964–1.398), respectively). Subsequently, subgroup analysis based on sex was performed. In boys, short sleep duration was significantly correlated with a high occurrence of obesity/overweight (odds ratio 1.294 (95% CI 1.153–1.452)) (Figure 2A). However, in the girls’ subgroup, there was no significant correlation between obesity/overweight and short sleep duration (Figure 2B).

Next, to evaluate appropriate sleep duration, a subgroup analysis based on sleep time was performed. Among various sleep times, the most common criterion was 10 h. In the 10 h subgroup, the odds ratio for obesity/overweight was 1.328 (95% CI 1.185–1.489) (Table 3). In addition, the odds ratios for obesity and overweight were 1.304 (95% CI 1.006–1.690) and 1.239 (95% CI 1.042–1.475), respectively. In the <6 and 6–10 years’ subgroups, there were significant correlations between short sleep duration and risk of obesity and overweight. In addition, significant correlations were found in the North American and Asian 10 h subgroups.

## 4. Discussion

The present investigation was a comprehensive systematic review and meta-analysis of the relationship between short sleep duration and the incidence of overweight and/or obesity among children and adolescents. Results reveal that short sleep duration increased the risk of overweight and/or obesity among children and adolescents, especially those <6 years and 6–10 years of age. Moreover, subgroup analyses revealed the difference of risk between geographical region, age, and sex.

Our study had several strengths. First, the odds ratio for obesity and overweight according to various sleep durations (from 5 h to 12 h in 1 h intervals) (data not shown) was investigated. Among various sleep times, the most common criterion was 10 h. In the 10 h subgroup, the odds ratio for obesity/overweight was 1.328 (95% CI 1.185–1.489) (Table 3). Second, the analysis investigated the odds ratio for obesity and overweight according to sleep time separately and in an integrated manner, and meaningful results were obtained. Third, in the current meta-analysis, we observed associations between increased age and obesity and overweight among children and adolescents according to short sleep duration, further reinforced by subgroup analysis according to age interval. We observed that short sleep duration may significantly increase the risk of overweight and/or obesity in the <6 and 6–10 years of age groups (Table 2). In addition, statistically significant results were obtained for boys and nonsignificant results for girls.

A study by Guo et al., reported that short sleep duration increased the risk of developing overweight or obesity by 35–41% among Chinese children and adolescents [34], which is consistent with our results. The association between short sleep duration and overweight/obesity varies according to age and sex. Several studies have investigated sleep duration and overweight/obesity in terms of sex [13,14]. For example, several studies have reported that the effect of sleep duration on obesity is an increased risk of obesity among women who sleep less [21,35,36]. Conversely, it was reported that girls had a lower risk of obesity due to short sleep duration than boys [16] and, although there was a difference according to age, it was reported that men had a higher risk of obesity due to shorter sleep duration than women [14]. In our study, shorter sleep duration was significantly correlated with a higher incidence of obesity/overweight among boys (odds ratio 1.294 (95% CI 1.153–1.452)); however, no such correlation was found among girls.

In the <6 years’ subgroup, the incidence of obesity/overweight was significantly increased in the <10 h group. In >10 years’ subgroup, the incidence of obesity/overweight was significantly increased in the <7 h subgroup. However, in the 6–10 years’ subgroup, statistical differences in the incidence of obesity/overweight were found at 8, 9, and 10 h. According to our results, recommendations for appropriate sleep duration to reduce obesity/overweight are different among various age groups.

The observation that long sleep time was associated with greater increases in adiposity is in line with some previous data [37,38]. In this regard, the first possibility is that long-duration sleepers are characterized by reduced energy expenditure due to increased time in bed. In addition, long-duration sleepers were more likely to gain weight during the study period because they had higher BMIs at baseline [39]. Another possibility pertains to the fact that obesity is associated with increased proinflammatory cytokines, which promote sleep [40].

In a subgroup analysis of sleep duration, Deng et al., reported that the objective method (actigraphy) demonstrated a greater effect on childhood obesity than subjective report [41]. In further studies, to determine the impact of overweight and obesity, objective measurement methods, such as actigraphy, rather than subjective reports, should be performed. In addition, the measurement of sleep duration should be more accurate and objective, taking into account naps, sleep quality, and other possible confounding factors that could affect sleep.

There were some limitations to this study that should be addressed. First, because this study was limited to North America and Asia, racial differences could not be compared; as such, further investigation is required. Second, we only considered sleep duration, while some important subgroup factors, such as naps, socioeconomic status, and other lifestyle factors, were not considered in all relevant studies. Third, long sleep time is an important factor in childhood and adolescent obesity and/or overweight; however, sufficient analysis has not been performed due to the lack of relevant data.

The present study provides evidence to the effect that both short and long sleep duration predict obesity/overweight. These results emphasize that sleep duration must now be considered as a new and potentially important determinant of obesity in current lifestyles.

In future studies, we will examine how sleep and obesity/overweight vary according to age and gender and prevent the development of obesity/overweight in individuals with either very long or very short sleep duration.

## 5. Conclusions

Our findings suggest that short sleep duration may increase the risk of obesity among children and adolescents, especially those <6 and 6–10 years of age, and long sleep duration did not yield any significant results. Odds ratio analysis of obesity/overweight for short sleep time revealed statistically significant results among Asians and boys.

## Figures and Tables

**Table 1:** Main characteristics of eligible studies.

First Author	Location	No of Patients	Age Group	Parameter	Comparison	Reference
Anujuo 2016	Various	2384	5 yrs	OS/OW	<10 h	>10 h
Azadbakht 2013	Iran	5528	10–18 yrs	OS, OW	<5 h	5–8 h
			10–14 yrs		<5 h	>8 h
Breitenstein 2019	USA	382	12–13 yrs	OS/OW	8 h	ND
Calamaro 2010	USA	13,568	15.96 yrs	OS	<6 h	8–11 h
					6–8 h	8–11 h
					8–11 h	11–14 h
Cao 2018	China	18,302	6–17 yrs	OW	<7 h	>9 h
					7–9 h	>9 h
Carrillo-Larco 2014	Various	1929	7.9 yrs	OS, OW	<10 h	10–11 h
Chaput 2006	Canada	422	5–10 yrs	OS/OW	8–10 h	12–13 h
					10.5–11.5 h	12–13 h
Chaput 2011	USA	550	9.6 yrs	OS/OW	<10 h	11–11.9 h
					10–10.9 h	11–11.9 h
					11–11.9 h	>12 h
Gong 2020	China	3411	12–13 yrs	OW	Short	ND
Ievers-Landis 2008	USA	819	9.5 yrs	OS		1 h reduction
Malihi 2021	New Zealand	5734	2 yrs	OS	<11.5 h	>11.5 h
Mercado-Gonzales 2019	Various	1945	4–5 yrs	OS	<10 h	10–13 h
Suglia 2013	USA	1589	5 yrs	OS	<9 h	>9 h
Suglia 2014	USA	10,076	16 yrs	OS	<6 h	>8 h
					6–8 h	>8 h
Sun 2009	Japan	5753	12–13 yrs	OW	<7 h	8–9 h
					7–8 h	8–9 h
					8–9 h	>9 h
Touchette 2008	Canada	2223	6 yrs	OS/OW	10 h	11 h
Wang 2016	Singapore	48,922	5 yrs	OS, OW	<10 h	11–12 h
					11–12 h	>13 h
Wang 2019	Hong Kong	3614	9–11 yrs	OS/OW	<9 h	>9 h
			11–16 yrs		<9 h	>9 h
Wing 2009	China	5159	9.25 yrs	OS/OW	9–10 h	>10 h
					8–9 h	>10 h
					<8 h	>10 h

yrs, years; OS, obesity; OW, overweight; h, hour

**Table 2:** Meta-analysis for the odds ratio of obesity/overweight according to the short sleep duration.

	Number of Subset	Fixed Effect (95% CI)	Heterogeneity Test (*p*-Value)	Random Effect (95% CI)	Egger’s Test
Obesity/overweight	54	1.169 [1.130, 1.210]	<0.001	1.171 [1.092, 1.256]	0.832
Obesity	17	1.225 [1.146, 1.310]	0.001	1.191 [1.055, 1.344]	0.294
Overweight	19	1.118 [1.068, 1.171]	<0.001	1.098 [0.976, 1.234]	0.669
<6 years	14	1.172 [1.119, 1.227]	<0.001	1.226 [1.083, 1.387]	0.516
6–10 years	9	1.341 [1.219, 1.474]	0.093	1.341 [1.175, 1.530]	0.771
>10 years	26	1.055 [0.980, 1.134]	<0.001	1.034 [0.904, 1.183]	0.489
North America	14	1.191 [1.089, 1.302]	<0.001	1.161 [0.964, 1.398]	0.587
Asia	30	1.158 [1.113, 1.206]	<0.001	1.161 [1.060, 1.271]	0.817

CI, confidence interval.

**Table 3:** Meta-analysis for the odds ratio of obesity/overweight according to the sleep time.

	Number of Subset	Fixed Effect (95% CI)	Heterogeneity Test (*p*-Value)	Random Effect (95% CI)	Egger’s Test
<10 h					
Obesity/overweight	14	1.212 [1.156, 1.270]	0.001	1.328 [1.185, 1.489]	0.094
Obesity	3	1.274 [1.149, 1.413]	0.040	1.304 [1.006, 1.690]	0.879
Overweight	3	1.169 [1.106, 1.236]	0.009	1.239 [1.042, 1.475]	0.535
<6 years	11	1.205 [1.147, 1.266]	0.001	1.326 [1.159, 1.518]	0.221
6–10 years	3	1.280 [1.095, 1.495]	0.094	1.365 [1.042, 1.789]	0.035
>10 years	0				
North America	2	1.966 [1.367, 2.826]	0.591	1.966 [1.367, 2.826]	-
Asia	5	1.203 [1.144, 1.265]	0.002	1.325 [1.154, 1.520]	0.038

CI, confidence interval.
